# *In vivo* antipyretic, antiemetic, *in vitro* membrane stabilization, antimicrobial, and cytotoxic activities of different extracts from *Spilanthes paniculata* leaves

**DOI:** 10.1186/0717-6287-47-45

**Published:** 2014-09-18

**Authors:** Mohammad Mobarak Hossain, Sayed Koushik Ahamed, Syed Masudur Rahman Dewan, Md Mahadi Hassan, Arif Istiaq, Mohammad Safiqul Islam, Md Mizanur Rahman Moghal

**Affiliations:** Department of Pharmacy, Noakhali Science and Technology University, Sonapur, Noakhali, 3814 Bangladesh; Department of Microbiology, Noakhali Science and Technology University, Sonapur, Noakhali, 3814 Bangladesh; Department of Life Science and Biotechnology, Faculty of Life and Environmental Science, Shimane University, 1060 Nishikawatsu-cho, Matsue-shi, Shimane, 690-8504 Japan

**Keywords:** Antiemetic, Anti-inflammatory, Antimicrobial activity, Brine shrimp lethality bioassay, Cytotoxic, Membrane stabilization, *Spilanthes paniculata*

## Abstract

**Background:**

The study was conducted to evaluate the *in vitro* antimicrobial activity, cytotoxic, and membrane stabilization activities, and *in vivo* antiemetic and antipyretic potentials of ethanolic extract, n-hexane and ethyl acetate soluble fractions of *Spilanthes paniculata* leaves for the first time widely used in the traditional treatments in Bangladesh.

**Results:**

In antipyretic activity assay, a significant reduction (*P* < 0.05) was observed in the temperature in the mice tested. At dose 400 mg/kg-body weight, the n-hexane soluble fraction showed the effect (36.7 ± 0.63°C ) as like as the standard (dose 150 mg/kg-body weight) after 5 h of administration. Extracts showed significant (*P* < 0.001) potential when tested for the antiemetic activity compared to the standard, metoclopramide. At dose 50 mg/kg-body weight, the standard showed 67.23% inhibition, whereas n-hexane and ethyl acetate soluble fractions showed 37.53% and 24.93% inhibition of emesis respectively at dose 400 mg/kg-body weight. In antimicrobial activity assay, the n-hexane soluble fraction (400 μg/disc) showed salient activity against the tested organisms. It exerts highest activity against *Salmonella typhi* (16.9 mm zone of inhibition); besides, crude, and ethyl acetate extracts showed resistance to *Bacillus cereus* and *Bacillus subtilis*, and *Vibrio cholera* respectively. All the extracts were tested for lysis of the erythrocytes. At the concentration of 1mg/ml, ethanol extract, and n-hexane and ethyl acetate soluble fractions significantly inhibited hypotonic solution induced lysis of the human red blood cell (HRBC) (27.406 ± 3.57, 46.034 ± 3.251, and 30.72 ± 5.679% respectively); where standard drug acetylsalicylic acid (concentration 0.1 mg/ml) showed 77.276 ± 0.321% inhibition. In case of heat induced HRBC hemolysis, the plant extracts also showed significant activity (34.21 ± 4.72, 21.81 ± 3.08, and 27.62 ± 8.79% inhibition respectively). In the brine shrimp lethality bioassay, the n-hexane fraction showed potent (LC_50_ value 48.978 μg/ml) activity, whereas ethyl acetate fraction showed mild (LC_50_ value 216.77 μg/ml) cytotoxic activity.

**Conclusions:**

Our results showed that the n-hexane extract has better effects than the other in all trials. In the context, it can be said that the leaves of *S. paniculata* possess remarkable pharmacological effects, and justify its folkloric use as antimicrobial, antipyretic, anti-inflammatory, and antiemetic agent. Therefore, further research may be suggested to find possible mode of action of the plant part.

## Background

Presently, drug resistance has become a serious global health problem, and spread of resistance poses additional challenges for clinicians and the pharmaceutical industries. Use of herbal drugs in the developed world continue to rise because they are rich source of novel drugs and their bioactive principles form the basis in medicine, nutraceuticals, pharmaceutical intermediates and lead compounds in synthetic drugs. Screening medicinal plants for biologically active compounds offers clues to develop newer antimicrobial agents [1]. Antiemetic agents are those which work against emesis induced by side effects of different drugs, general anesthetics, chemotherapeutic agents, and motion sickness [2]. Hyperpyrexia or fever is usually caused as a secondary impact of infection, tissue damage, inflammation, graft rejection and malignant tumors or other diseased states. Typically, the infected or damaged tissue initiates increased formation of pro-inflammatory mediators, including cytokines such as interleukin 1β, α, β and TNF-α, which generally increase the synthesis of prostaglandin E_2_ (PGE_2_) near preoptic hypothalamus area and thus triggering the hypothalamus to elevate the body temperature [3]. Membrane stabilization is a possible mechanism of action for the anti-inflammatory activity. There are many anti-inflammatory drugs, such as nonsteroidal anti-inflammatory drugs (NSAIDs) to treat the consequences of inflammation. The effect of these drugs including herbal preparation on the stabilization of erythrocyte membrane exposed to hypotonic and heat has been studied extensively. But these studies showed that, these drugs are not free from adverse effects, as they are responsible for intestinal side effects and mucosal erosions that can progress into ulcers [4]. For these reasons, many researchers have focused on medicinal plants for finding natural anti-inflammatory drugs.

*S. paniculata* belongs to the family Asteraceae, commonly known as toothache plant, or *Shormoni* (Bengali). The various synonyms of the plant are *Bidens acmella, Bidens ocymifolia, Pyrethrum acmella, Spilanthes ocymifolia, Verbesina ocymifolia,* etc. The raw leaves of *S. paniculata* are used as flavouring agents for salads, soups and meats in Brazil and India. It is grown widely as an ornamental plant because of the attractive colorful heads. *S. paniculata* all showed larvicidal activity against *Anopheles* mosquitoes suggesting a possible role for *Spilanthes* in not just the treatment but also prevention of malaria [5]. *Spilanthes* contains a number of biologically active compounds [6], of which the most studied have been the alkylamides [7]. Isolated alkylamides from *Spilanthes* have demonstrated activity against mosquito larvae. Although there are no published reports of antiplasmodial activity of isolated *Spilanthes* alkylamides, but show such activity from other plants have [8]. Roots of *S. paniculata* release more than 90% of N, P and K within 150 days. *S. paniculata* can play a significant role in soil nutrient enrichment in poorly managed shifting cultivation systems [9]. In addition, in 2012, Hossain and his colleagues exerted a report on the leaves of this plant. They showed that the leaves conserve antinociceptive, antioxidant, and anti-inflammatory (on mice) activities [10, 11]. The tests which have been carried out in the present study have not been conducted before on the leaves of this plant native to Bangladesh, though antimicrobial and cytotoxic assays have been carried out with the whole plant [12]; therefore, we chose the plant. In this study, our main goal was to evaluate antiemetic, antipyretic, and *in vitro* membrane stabilizing (using human red blood cell -RBC), cytotoxic and antimicrobial activities of *S. paniculata* to validate its use in traditional treatments.

## Results

### Antipyretic activity test

Effect of different extracts of *S. paniculata* on rectal temperature in mice is presented in Table 1. The subcutaneous injection of yeast suspension markedly elevated the rectal temperature after 18 h of administration. Treatment with n-hexane extract showed significant activity against induced pyrexia when compared with the control treatment.

**Table 1:** **Effect of the crude, n-hexane, and ethyl acetate extracts of** ***S. paniculata*** **on yeast-induced pyrexia in mice**

Treatment	Dose mg/kg	Mean ± SEM rectal temperature (°C)					
		0 h	1 h	2 h	3 h	4 h	5 h
Control	-	37.4 ± 0.5	36.9 ± 0.5	37.03 ± 0.12	37.07 ± 0.12	37.03 ± 0.13	36.9 ± 0.7
Standard (Paracetamol)	150	37.2 ± 0.1	36.5 ± 0.6**	36.8 ± 0.26	36.77 ± 0.18**	36.9 ± 0.31	36.7 ± 0.70**
Crude extract	400	37.03 ± 0.2	36.8 ± 0.6	37.2 ± 0.07	36.93 ± 0.15	37 ± 0.26	36.8 ± 0.61
n-hexane extract	400	36.9 ± 0.4	36.7 ± 0.6**	36.7 ± 0.37	36.77 ± 0.12**	36.82 ± 0.41	36.7 ± 0.63**
Ethyl acetate extract	400	37 ± 0.3	36.8 ± 0.6	36.8 ± 0.26	37.03 ± 0.24	37.1 ± 0.23	36.8 ± 0.63

Here, **Significant difference at *P* < 0.05 vs. control (n = 5); SEM = standard error mean.

### Antiemetic activity test

Result of the antiemetic activity of the extracts of *S. paniculata* leaves is given in Table 2. After administration of a dose of 50 mg/kg-body weight metoclopramide and the extracts of leaves, the numbers of retches were reduced. On the basis of these results, it is stated that n-hexane and ethyl acetate extracts of *S. paniculata* leaves have significant antiemetic potential which are comparable with that of metoclopramide (standard drug). Although, the results are significant (*P* < 0.001), the mode of action is still unknown.

**Table 2:** **Antiemetic activities of ethanolic extract and fractions of** ***S. paniculata*** **leaves**

Treatments	Doses	Mean number of retches ± SEM	% inhibition of emesis
Control	10 ml/kg	71.4 ± 1.76	**-**
Standard	50 mg/kg	23.4 ± 2.85	67.23
Crude extract	200 mg/kg	46.2 ± 1.80	39.78
	400 mg/kg	44.6 ± 2.10	37.54
n-hexane extract	200 mg/kg	46.8 ± 2.02	34.45**
	400 mg/kg	44.6 ± 2.03	37.53**
Ethyl acetate extract	200 mg/kg	54.4 ± 1.86	23.80**
	400 mg/kg	53.6 ± 2.07	24.93**

Here, **Significant difference at *P* < 0.001 vs. control; SEM = standard error mean.

### Antimicrobial activity test

**Figure 1:**
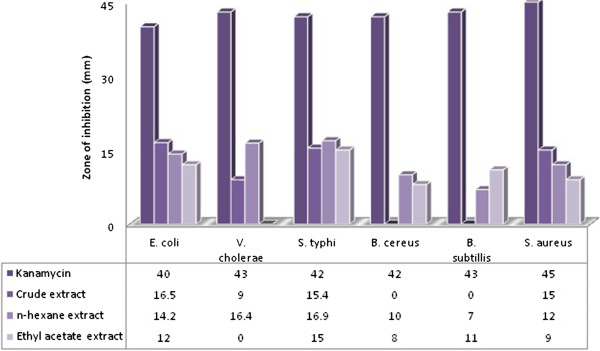
**Antimicrobial activity of test samples of** ***S. paniculata.***

Among the all plant extractives, n-hexane extract showed highest activity against *S. typhi*, and also expressible potential against almost all the test organisms (Figure 1). The zones of inhibition of *S. paniculata* were low in comparison with the standard kanamycin; therefore, the MIC (minimum inhibitory concentration) was not determined.

### Membrane stabilizing activity

The crude ethanol extract of leaves of *S. paniculata*, as well as different partitionates derived from this extract, were subjected to assay for membrane stabilizing activities following standard protocols and the obtained results were statistically represented in Tables 3 and 4. The results showed that the extracts (at concentration 1 mg/ml) were significantly potent on human erythrocyte, adequately protecting it against hypotonic solution and heat induced lyses, when compared with the standard drug acetylsalicylic acid (0.10 mg/ml).

**Table 3:** **Hypotonic solution induced hemolysis of erythrocyte membrane**

Treatment	Concentration	Mean ± SD hypotonic solution	% of inhibition of hemolysis ± SEM
Control	1 mg/ml	0.695 ± 0.0025	**-**
Crude	1 mg/ml	0.505 ± 0.041*	27.406 ± 3.57**
n-hexane	1 mg/ml	0.375 ± 0.04**	46.034 ± 3.251*
Ethyl acetate	1 mg/ml	0.482 ± 0.068*	30.72 ± 5.679*
Acetly salicylic acid	0.10 mg/ml	0.158 ± 0.041**	77.276 ± 0.321

Level of significance **P < 0.001, *P < 0.01 percent inhibition of migration was calculated relative to control.

**Table 4:** **Heat induced hemolysis of erythrocyte membrane**

Treatment	Concentration	Optical density of sample		% Inhibition of haemolysis
		Heated solution	Unheated solution	
Control	1 mg/ml	0.744 ± 0.065	0.352 ± 0.21	**-**
Crude extract	1 mg/ml	0.51 ± 0.0197*	0.39 ± 0.074	34.21 ± 4.72*
N-Hexane	1 mg/ml	0.43 ± 0.06*	0.35 ± 0.079	21.81 ± 3.08**
Ethyl acetate	1 mg/ml	0.476 ± 0.049*	0.37 ± 0.11	27.62 ± 8.79
Acetylsalicylic acid	0.10 mg/ml	0.64 ± 0.071	0.41 ± 0.12	70.05 ± 3.87

Level of significance **P < 0.001, *P < 0.01 percent inhibition of migration was calculated relative to control.

### Brine shrimp lethality bioassay

LC_50_ (lethal concentration of half of the test organisms) data of vincristine sulphate, and n-hexane and ethyl acetate extracts have been given in Table 5.

**Table 5:** **Cytotoxic effects of leaves of** ***S. paniculata***

Sample	LC_50_(μg/ml)	Regression equation	R^2^
Vincristine sulphate (positive control)	0.563	y = 30.056x + 56.016	0.9168
n-Hexane soluble fractions	48.978	y = 42.887x – 22.502	0.671
Ethyl acetate soluble fraction	216.770	y = 29.397x -18.673	0.4908

## Discussion

It is evidence that the ethanolic extract and its different fractions protected the human erythrocyte membrane against lysis induced by hypotonic solution and heat. During inflammation, lysosomal enzymes and hydrolytic components are released from the phagocytes to the extracellular space, which causes damages of the surrounding organelles and tissues and also assists a variety of disorders [13]. It was found that NSAIDs act either by inhibiting these lysosomal enzymes or through stabilization of lysosomal membranes. Again, RBC exposure to harmful substances such as hypotonic medium, heat, etc results in the lysis of the membranes, accompanied by the oxidation and the lysis of hemoglobin [14]. The inhibition of hypotonicity and heat induced RBC membrane lysis was taken as a measure of the mechanism of anti-inflammatory activity of the plant extract, because human RBC membranes are considered similar to lysosomal membrane components [15]. One can say that the possible mode of action of the extract, fractions and standard anti-inflammatory drugs may be connected with binding to the erythrocyte membranes with consequent alteration of surface charges of cells. This could have prevented physical interaction with agents of aggregation or promote dispersion by mutual repulsion of the charges as being involved in the hemolysis of RBCs. In some research, it has been reported that some chemical components present in the extracts can have the same mechanism, which are well known for their anti-inflammatory activity [16]. Both *in vitro* and *in vivo* studies in experimental animals showed that the flavonoids exert stabilizing effects largely on lysosomes [17] as tannin and saponins are capable of binding cations and other biomolecules, and are capable of stabilizing the erythrocyte membrane [18]; and report says that the leaf extract of *S. paniculata* has tannins, saponin, and lots of flavonoids [11]. Our research reveals that all the extracts showed potent RBC membrane stabilization activity with a good protection against both hypotonic solution and heat-induced lysis. In the hypotonic solution induced hemolysis, the n-hexane soluble fraction, and in heat induced hemolysis, the crude extract, were found having better activity.

Subcutaneous administration of Brewer’s yeast induces pyrexia by increasing the synthesis of prostaglandin. It is considered as a useful experiment for the screening of plants materials as well as synthetic drugs for their antipyretic effect [19]. Yeast-induced pyrexia is known as pathogenic fever. Its etiology could be the production of prostaglandins [20]. The inhibition of prostaglandin production could be the possible mechanism of antipyretic action as that of paracetamol and it can be achieved by blocking the cyclooxygenase enzyme activity. There are several mediators in our body for pyrexia and the proper inhibition of these mediators is more responsible for the antipyretic effect [21]. The intraperitoneal administration of different leaves-extract significantly attenuated rectal temperature of yeast induced febrile mice. Thus it can be postulated that the extract contained pharmacologically active principle(s) that interfere with the release of prostaglandins. Thus this result supports the use of *S. paniculata* as an antipyretic agent for the treatment of fever.

For the development of antimicrobial agents, plants are important sources of potentially useful structures, because they are available, therefore profitable [22]; thus *in vitro* antibacterial activity assay is the preliminary step towards this goal. The microorganisms (bacteria and fungi) used in the present study were selected for their clinical importance as they frequently cause resistance against different antibiotics [23]. While screening medicinal plants for antibacterial activity it is generally expected that a greater number of compounds would be active against broad spectrum of microorganisms [24]. Again, it is known that phytochemicals like flavonoids, tannins, saponin and some other phenolic compounds are responsible for the antimicrobial activity of a plant extract [25], and it has been already reported that this plant possesses these significantly. However, in our study it was found that all the extracts showed remarkable antimicrobial activity; where the crude extract showed resistance against *B. cereus* and *B. subtilis*, although in another study in 2011, they reported that the plant extract showed activity against these microbes [12]. It can be due to the different source of organism or the plant part. They used the whole plant for the study. Therefore, it is clear from the study that whatever the mechanism is, some of these phytocomponents may be responsible for the potent antimicrobial activity of the plant.

Reports say that the oral copper sulfate induces emesis acting on the peripheral nervous system [26] and the peripheral 5- HT_4_ play an important role in this action [27]. The standard drug metoclopramide exerts its antiemetic effect through acceleration of gastrointestinal tract movement [28] which was found more effective than the leaf extracts. Again, several studies were carried out to identify the responsible phytochemicals for the antiemetic activity. These studies reported that several active compounds like flavonoids, terpenes, alkaloids etc. may be involved in the antiemetic activity of plant extracts [29]. We found that the crude ethanolic extract is abundant of flavonoids, tannin, and saponin [11], and these compounds may be responsible for the possible antiemetic activity, although the exact mode of action is yet to be discovered. Plants are important resources for the development of new chemotherapeutic agents. One of the most important steps toward this goal is the brine shrimp lethality bioassay (BSLB). It is used widely in the preliminary screening of the crude extracts as well the fractions, and isolated compounds to evaluate the toxicity towards brine shrimps, which may also provide an indication of possible cytotoxic properties of the test materials [30]. The method is easy to conduct and it is said that the cytotoxic compounds generally exhibit significant activity in the BSLB; therefore this assay can be recommended as a guide for the detection of antitumour compounds and pesticides due to its low cost [31]. This bioassay has also a good correlation with the human solid tumour cell lines. Therefore the cytotoxic effects of the plant extracts enunciate that it can be selected for further cell line assay due to the correlation between cytotoxicity and activity against the brine shrimp nauplii using extracts [32]. Hence, the present study supports BSLB as a reliable method for the assessment of bioactivity of Bangladeshi medicinal plant, *S. paniculata* and lends support for their use in pharmacology. In this study, the ethyl acetate extract showed slight cytotoxic potential, whereas the n-hexane soluble extract showed potent activity as the report of Anderson *et al*. [33] says that the significant lethality (as LC_50_ value less than 100 ppm or μg/ml) of the plant extract to brine shrimp is indicative of the presence of potent cytotoxic and probably insecticidal compounds which warrants further investigations.

## Conclusions

In light of the results of this study, it can be summarized that the plant extracts have noteworthy anti-inflammatory property, antiemetic, antipyretic, cytotoxic and broad-spectrum antimicrobial activities. Therefore, it may suggest further studies to better understand the mechanism of such actions scientifically.

## Methods

### Plant material collection and identification

For the investigation, the leaves of *S. paniculata* were collected by the authors from the surrounding area of Noakhali, Bangladesh in July 2012. The plant was identified and authenticated by an expert botanist of Bangladesh National Herbarium (DACB), Mirpur, Dhaka (Accession No. 39538) and a voucher specimen was submitted at the herbarium for future reference.

### Extract preparation

Weighed (600 g of the dried and powdered) sample was soaked in 1500 ml of 80% ethanol (Merck KGaA, Germany) in clean, sterilized, and flat-bottomed glass container. Afterwards, it was sealed and maintained for 15 days accompanying occasional stirring and agitation. The complete mixture was then subjected to coarse filtration on a piece of clean, white sterilized cotton material and Whatman® filter paper. The extract was obtained by evaporation using rotary evaporator (Bibby RE-200, Sterilin Ltd., UK) at 4 rpm and 65°C temperature. It rendered a gummy concentrate of greenish black color. The gummy concentrate was designated as crude extract, or ethanolic extract. Then the crude ethanolic extract was dried by freeze drier and preserved at +4°C (yield 16.55%). After that, 15 g of crude extract was triturated with 270 ml of ethanol containing 30 ml distilled water. The crude extract was dissolved completely to obtain the mother solution. This solution was partitioned successfully two solvents of different polarity. The mother solution was taken in a separating funnel. 100 ml of n-hexane was added here, and the funnel was shaken and kept undisturbed. Then the organic portion was collected and repeated thrice. Ethyl acetate extract was collected with the help of aqueous mother fraction adding 55 ml of distilled water keeping the other procedure unchanged. Finally, n-hexane, and ethyl acetate extracts were found and preserved for the next steps.

### Chemicals

All the chemicals used in this study were of analytical grade, and purchased from Sigma Chemical Co. (St. Louis, MO, USA), and Merck (Darmstadt, Germany).

### Test animals

For the screening of *in vivo* antipyretic potential of *S. paniculata* leaves, young Swiss-albino mice (aged 20-25 days) of either sex, average weight 20-25 g were used. They were collected from the Animal Resources Branch of ICDDR, B (International Centre for Diarrheal Disease and Research, Bangladesh). After collection, they were kept in favorable condition for one week for adaptation and fed rodent food and water *ad libitum* formulated by ICDDR, B. For *in vivo* antiemetic activity test, young chicks of either sex (aged 2- 4 days), weighing from 32-52 g were obtained from a local poultry store. All chicks were kept under laboratory conditions at room temperature with 12 h light and dark cycles. Throughout the experiments, all animals received human care according to the criteria outlined in the ‘Guide for the Care and Use of Laboratory Animals’, 8th edition, prepared by the National Academy of Sciences and published by the National Institute of Health (US).

### Test organisms

Three strains of Gram-positive (*Bacillus cereus* ATCC 17549*, Bacillus subtilis* ATCC 8354*,* and *Staphylococcus aureus* ATCC 6538), and three strains of Gram-negative bacteria (*Escherichia coli* ATCC 8739*, Salmonella typhi* ATCC 14028*,* and *Vibrio cholera* ATCC 8027) were used to evaluate the antimicrobial activity. The organisms were sub-cultured in nutrient broth and nutrient agar. They were collected from the Department of Microbiology, Noakhali Science and Technology University, Bangladesh.

### Collection of blood samples

Human RBCs were collected for the study. 7 ml of blood was collected from each of the healthy Bangladeshi human volunteers (aged 20-23 years) without a history of oral contraceptive or anticoagulant therapy and free from diseases (using a protocol approved by Ethical Committee of NSTU Research Cell, Noakhali Science and Technology University). The collected RBCs were kept in a test tube with an anticoagulant EDTA under standard conditions of temperature 23 ± 2°C and relative humidity 55 ± 10%.

### Antipyretic activity test

The antipyretic activity was evaluated by Brewer’s yeast induced pyrexia in experimental animal [33]. Hyperpyrexia was induced by subcutaneous administration of 10 ml/kg body weight 20% aqueous suspension of brewer’s yeast. The selected animals were fasted overnight with water *ad libitum* before the experiments. Initial rectal temperatures of the animals were recorded using an Ellab thermometer. After 18 h of subcutaneous administration the animals that showed an increase of 0.3–0.5°C in rectal temperature were selected for the antipyretic activity. Different extracts of plant were given orally (400 mg/kg-body weight). Paracetamol (150 mg/kg-body weight orally) was used as reference drug. Control group received distilled water (10 ml/kg) only. The rectal temperature was recorded at 1 h intervals for 4 h after treatment [34].

### *In vivo*antiemetic activity test

The chicks were divided into seven groups of five chicks each and each chick was kept in a large beaker at room temperature for 10 min. The antiemetic effect was determined by calculating the mean decrease in number of retching following the protocols reported by Akita *et al.* [35]. The crude and fractionate extracts of *S. paniculata* leaves were dissolved in 0.9% saline containing 5% DMSO and 1% Tween 80 and administered at doses of 200 and 400 mg/kg-body weight orally and volume of 10 ml/kg-body weight to the test animal on the basis of their body weights. Control group received only saline 0.9%. After 10 min, copper sulphate was administered orally at 50 mg/kg-body weight, then the number of retching was observed during next 10 min. Metoclopramide was used as a standard drug (50 mg per kg body weight) intraperitoneally. The antiemetic effect was assessed as the decrease in number of retches in the treated group in contrast to the control. The inhibition (%) was calculated by the following equation:

Inhibition (%) = ((*A*−*B*)/*A*) × 100

Where, A is the control frequency of retching and B is the frequency of retching of the treated groups.

### Disc diffusion assay (DDA)

Disc diffusion method is widely acceptable for the evaluation of antimicrobial activity [36]. In this method, an antibiotic was diffused from a reliable source through the nutrient agar and a concentration gradient was created. Dried, sterilized filter paper discs (6 mm diameter, HI-Media, China) containing the known concentration of test samples (400 μg/disc) were placed on nutrient agar medium consistently seeded with the test organisms. As positive and negative control, standard antibiotic of kanamycin (30 μg/disc) and blank discs were used. For the maximum diffusion of the test materials to the surrounding media these plates were reserved at low temperature (4°C) for 24 h. The plates were then incubated at 37°C for 24 h to allow optimum growth of the microbes. The test materials having antimicrobial property inhibit microbial growth in plates and thereby yield a clear, distinct zone defined as zone of inhibition. The activity of the test sample was then determined by measuring the zone of inhibition expressed in millimeter [37].

### Assay of membrane stabilization

#### Erythrocyte suspension

The blood was washed three times using isotonic solution (0.9% saline). The volume of saline was measured and reconstituted as a 40% (v/v) suspension with isotonic buffer solution (pH 7.4) which contained in 1 L of distilled water: NaH_2_PO_4_. 2H_2_O, 0.26 g; Na_2_HPO_4_, 1.15 g; NaCl, 9 g (10 mM sodium phosphate buffer). Thus the suspension finally collected was the stock erythrocyte (RBC) suspension.

#### Hypotonic solution induced hemolysis

The membrane stabilizing activity of the extracts was evaluated by using hypotonic solution induced human erythrocyte hemolysis, designed by Sikder *et al*. [38] with minor modification. To prepare the erythrocyte suspension, blood (7 ml) was obtained using syringes (containing anticoagulant EDTA) from male volunteers through puncture of the anti-cubital vein. The blood was centrifuged, using centrifugal machine, for 10 min at 3000 g and blood cells were washed three times with solution (154 mM NaCl) in 10 mM sodium phosphate buffer (pH 7.4). The test sample, consisted of stock erythrocyte (RBC) suspension (0.50 ml), was mixed with 5 ml of hypotonic solution (5 mM NaCl) in 10 mM sodium phosphate buffered saline (pH 7.4) containing either the extracts (1.0 mg/ml) or acetylsalicylic acid (0.1 mg/ml). The control sample, consisted of 0.5 ml of RBCs, was mixed with hypotonic-buffered saline alone.

The mixture was incubated for 10 min at room temperature, centrifuged for 10 min at 3000 g and the absorbance of the supernatant was measured at 540 nm using UV spectrophotometer (Shimadzu, Japan). The percentage inhibition of either hemolysis or membrane stabilization was calculated using the following equation:

% inhibition of hemolysis = 100 X (OD_1_- OD_2_/OD_1_)

Where,

OD_1_ = Optical density of hypotonic-buffered saline solution alone (control) and,

OD_2_ = Optical density of test sample in hypotonic solution.

#### Heat-induced hemolysis

Aliquots (5 ml) of the isotonic buffer, containing 1.0 mg/ml of different extracts of the plant were put into two duplicate sets of centrifuge tubes [39]. The vehicle, in the same amount, was added to another tube as control. Erythrocyte suspension (30 mL) was added to each tube and mixed gently by inversion. One pair of the tubes was incubated at 54°C for 20 min in a water bath. The other pair was maintained at 0-5°C in an ice bath. The reaction mixture was centrifuged for 3 min at 1300 g and the absorbance of the supernatant was measured at 540 nm using UV spectrometer. The percentage inhibition or acceleration of hemolysis in tests and was calculated using the following equation:

% inhibition of hemolysis = 100 × [1-(OD_2_-OD_1_/OD_3_-OD_1_)]

Where,

OD_1_ = test sample unheated,

OD_2_ = test sample heated and,

OD_3_ = control sample heated.

#### Brine shrimp lethality bioassay

The cytotoxic activities of the extracts were examined using brine shrimp lethality bioassay [30]. *Artemis salina* leach (brine shrimp eggs) collected from pet shops was used as the test organism. Seawater was taken in the small tank and shrimp eggs were added to one side of the tank and then this side was covered. 24 h were allowed to hatch the shrimp and to be matured as nauplii. Constant oxygen supply was provided throughout the hatching time. The hatched shrimps were attracted to the lamp through the perforated dam. In this study vincristine sulphate was used as the positive control. Measured amount of the vincristine sulphate was dissolved in DMSO to get an initial concentration of 40 μg/ml from which serial dilutions were made using DMSO to get 20 μg/ml, 10 μg/ml, 5 μg/ml, 2.5 μg/ml, 1.25 μg/ml, 0.625 μg/ml, 0.3125 μg/ml, 0.15625 μg/ml and 0.78125 μg/ml solution from the extracts. Then the positive control solutions were added to the pre-marked vials containing ten living brine shrimp nauplii in 5 ml simulated sea water to get the positive control groups. 100 μl of DMSO was added to each of three pre-marked glass vials containing 5 ml of simulated sea water and 10 shrimp nauplii to use as control groups.

#### Counting of nauplii

After 24 h, by using a magnifying glass, the vials were inspected and the number of survived nauplii in each vial was counted. From this data, the percent (%) of lethality of the brine shrimp nauplii was calculated for each concentration.

#### Statistical analysis

One way ANOVA with Dunnett’s post Hoc test for this experiment was carried out with SPSS 16.0 for Windows® software and the results obtained were compared with the control group. *P* values < 0.05 were considered to be statistically significant.
